# Pediatric Fibrous Dysplasia of the Skull Base: Update on Management and Treatment

**DOI:** 10.3390/brainsci14121210

**Published:** 2024-11-29

**Authors:** Pierce Spencer, Vidhatri Raturi, Amanda Watters, R. Shane Tubbs

**Affiliations:** 1 Department of Neurological Surgery, Tulane University School of Medicine, New Orleans, LA 70112, USA; pspencer1@tulane.edu; 2 Tulane University School of Medicine, New Orleans, LA 70112, USA; vraturi@tulane.edu (V.R.); awatters@tulane.edu (A.W.)

**Keywords:** fibrous dysplasia, polyostotic, pediatric skull base lesions, fibro-osseous lesions, pediatric neurosurgery

## Abstract

Background: Fibrous dysplasia (FD) is often difficult for skull base surgeons to address. FD arises due to the abnormal proliferation of fibroblasts, ultimately resulting in immature osseous tissue replacing normal cancellous bone. When the skull base is involved, it can result in cranial nerve compression. FD affecting the optic canal and optic nerve is the most concerning as new onset of vision loss is considered a surgical emergency. The prevalence of FD is approximately 3.6 per 1,000,000. The most severe implications of this disease are neurological deficits due to cranial nerve compression, cosmetic appearance, and high recurrence rates even in the setting of surgical and medical therapy interventions. Methods: A PubMed search of “pediatric fibrous dysplasia management” using MESH Terms was conducted. Articles were excluded for non-English languages, inaccessibility, and events/erratum/letters to the editor. Included articles were in English, as well as encompassed pediatric FD case reports or comprehensive reviews of FD that discussed pediatric presentations. Results: A total of 109 articles were reviewed, and 44 were included in the final review. Most articles were case reports. There is a clear need for guidelines regarding surgical intervention, especially in the pediatric population, where hormonal fluctuation can influence rates of recurrence and bony deformity. Overall, most surgeons recommend close observation with biomarkers and radiographic imaging for asymptomatic patients until at least the age of 16 years old. Conservative methods, such as RANK-L inhibitors, can be utilized to decrease growth with some success, especially in older adolescents. Conclusion: This review is an update on this disease and its presentations, imaging findings, and treatment options. The current literature lacks clear guidance on management, especially in regard to surgical intervention or recurrence monitoring algorithms.

## 1. Introduction

Fibrous dysplasia is a benign genetic developmental disorder in which abnormal fibrous tissue intertwined with bone replaces normal bone and marrow. The woven bone fails to transform into lamellar bone, leading to highly vascularized fibrous stroma around abnormally arranged osseous trabeculae [1]. Consequently, the area of fibrous tissue interwoven with bone becomes soft and stringy in contrast to the hardness of normal bone. This can occur in any bone in the body and can be categorized as monostotic, polyostotic, or a component of the McCune-Albright syndrome [1,2,3].

The responsible GNAS gene, located on chromosome 20q13.3, encodes the alpha subunit of the stimulatory G protein of adenylyl cyclase, which stimulates cAMP formation. Activating mutations in this gene are the mechanism underlying fibrous dysplasia [3,4,5]. These missense post-zygotic mutations lead to abnormal proliferation of fibroblasts, ultimately resulting in immature osseous tissue replacing normal cancellous bone [6]. In craniofacial bones, the GNAS mutation disrupts normal skeletal stem cell function by inducing abnormal differentiation. This results in them overgrowing and transforming into fibroblasts rather than osteoblasts, ultimately leading to the formation of fibrous tissue and irregular bone structure. [7,8] The overall incidence of fibrous dysplasia is low, yet craniofacial involvement exists in up to 27% of monostatic cases and 50% of polyostotic cases [6,9]. The polyostotic state appears to be around four times more prevalent than monostotic [10]. McCune-Albright syndrome is the most uncommon form, making up about 5% of fibrous dysplasia cases with incidence rates as low as 1/100,000 to 1/1,000,000 [11]. A review by Yang et al. (2017) reported that among 487 patients with craniofacial fibrous dysplasia, monostotic, polyostotic, and McCune-Albright syndrome made up 56%, 47%, and 7%, respectively [12]. 

Of particular concern with FD is the high rate of recurrence, especially in pediatric populations who have had surgical intervention prior to the age of 16. A 2020 review of 33 patients with craniofacial involvement demonstrated a significant reduction in recurrence in patients older than the age of 16 with surgical intervention [13]. Another review of 133 subjects managed by plastic surgery found that the mean age to surgical intervention was 13.5 years of age, with craniofacial deformity being the leading cause of intervention (59%) and a 41% rate of recurrence in the 33 subjects who had surgical intervention [14]. Overall, McCune-Albright syndrome (88%), excess growth hormone (58%), and debulking procedures (82%) were significantly associated with recurrence (*p* = 0.02) [14]. However, aggressive reconstruction was shown to be significantly associated with reduced rates of recurrence (*p* = 0.007) [14]. The complexity of treatment depends on the individual needs of the patients, including considerations such as age, underlying mutations, disease category, and rarity of presentation. Additionally, cranial nerve involvement is a critical factor, particularly the optic, vestibular, cochlear, and facial nerves, which can lead to lifelong deficits such as blindness, hearing loss, and facial pain. The gold standard treatment for FD remains surgical intervention. However, in cases where hormone excesses are identified, there is a unique role for utilizing biomarkers and imaging for diagnosis, monitoring, and medical intervention to delay surgery [15]. 

Beyond the challenges in management mentioned above, there remains a clear lack of guidelines regarding intervention and monitoring for recurrence. We briefly outline craniofacial involvement of pediatric FD, including current diagnostics and interventions, as well as provide a broad overview of the current literature. We aim to not only highlight the current consensus amongst surgeons who operate on these complex pediatric skull-based bony lesions, but also comment on current trends, changes for radiographic monitoring, and medical therapeutic intervention in lieu of surgery where applicable. 

### 1.1. Craniofacial Bone Involvement

The base of the skull is a common location for fibrous dysplasia in the craniofacial region [16,17,18,19,20]. The zygomatic-maxillary complex is the most commonly affected region in monostotic FD. A systematic review of 487 patients with craniofacial fibrous dysplasia demonstrated that the bony regions most commonly affected in monostotic cases were the maxillary (28%), orbit (27%), mandible (25%), frontal (22%), and temporal (12%) [12].

In contrast, the anterior skull base (95% of cases) and the craniofacial region (90%) are typically more involved in polyostotic or McCune-Albright syndrome cases [21,22]. Up to 30% of polyostotic cases involved the maxilla [12]. A 2018 study of pediatric patients with either McCune-Albright syndrome or polyostotic fibrous dysplasia showed that seven (44%) cases involved the sphenoid bone, six (38%) the frontal bone, four (25%) the temporal bone, three (19%) the occipital bone, and two (13%) the ethmoid bone [23].

A 2022 study of 23 adults and pediatric patients with fibrous dysplasia impacting the air sinuses and skull base showed that 14 (61%) cases involved the sphenoid, nine (39%) the ethmoid, and two (9%) the frontal bone [6]. Lesions are more common in the anterior fossa, followed by the middle and posterior fossae [24]. Overall, amongst both monostotic and polyostotic populations, concern is typically most significant when the bone involved is the ethmoid, sphenoid, or maxilla because of cranial nerve deficits, such as optic nerve compression, facial nerve dysfunction, and vestibulocochlear nerve involvement, leading to hearing loss or balance problems [1,9] (Table 1).

### 1.2. Presentation

The condition often presents during adolescence due to rapid growth during puberty. Over 90% of lesions appear by the age of 15 [25]. Patients commonly present with insidious onset of symptoms, such as nasal obstruction, loss of vision, painless facial swelling, or asymmetry [9,25]. Patients with craniofacial bony involvement typically present earlier, with the median onset at age 3.4 [25]. The lesions are typically slow-growing and not usually a functional issue but more so a cosmetic concern [1,9,25]. Most lesions are benign; malignant transformation is rare and usually occurs in polyostotic rather than monostotic cases [9,24,25,26]. Monostotic patients typically present with fewer symptoms than their polyostotic counterparts or are even asymptomatic [25]. The most common presenting symptoms in both patient populations include facial pain, headache, sinus-related symptoms, ocular symptoms, palpable expanding masses, hearing loss, and facial numbness [1,9,24,25,26,27].

Facial asymmetry is the most common symptom among pediatric patients with FD and often appears early in the disease course. When the maxillary, mandibular, frontal, or zygomatic bones are involved, patients may develop a painless and slow-growing mass [28,29]. Sinonasal involvement has been described in anywhere from 36–92% of FD patients, with the most commonly affected locations being the frontal, maxillary, and ethmoid sinuses [30,31]. Although sinonasal involvement is typically asymptomatic, patients may experience symptoms such as rhinorrhea, nasal congestion or obstruction, pain, sinusitis, or headaches [32]. When orbital bones are involved, patients can present with proptosis, hypertelorism, diplopia, and other visual disturbances [33,34]. For up to 30% of patients, temporal bone involvement may lead to mild to moderate hearing loss [35,36]. When the mandible or maxilla is involved, there may be dental displacement or malocclusion [29,37]. Skull base involvement can narrow the optic canal, occasionally resulting in optic neuropathy [38]. FD within the posterior cranial fossa may cause cranial constriction or settling, ultimately resulting in skull base deformities such as Chiari malformation or basilar invagination [39]. Hyperthyroidism and hypophosphatemia are correlated with an increased risk of skull base deformities [32]. 

### 1.3. Diagnostics 

Asymptomatic patients with fibrous dysplasia are often diagnosed incidentally on imaging studies for other concerns, or go undiagnosed [9,40,41]. X-ray imaging is commonly used to monitor the progression of lesions, which appear dense and sclerotic [42]. CT is the gold standard for diagnosing fibrous dysplasia. It allows for adequate delineation of morphological bone changes, as well as for lesions to be better defined in relation to neuronal, vascular, and soft tissue structures [42]. With age, lesions become more heterogeneous, morphing from a homogenous “ground glass” appearance into discrete radiolucent, cystic-looking areas [42]. On magnetic resonance imaging (MRI), the degree of contrast enhancement depends on the number and level of osseous trabeculae, degree of cellularity, collagen content, and cystic and hemorrhagic changes [42]. The lesions often have explicitly differentiated borders, giving intermediate signal intensity on T1-weighted MRI and heterogeneous hypointense/intermediate signal intensity on T2-weighted MRI [42]. MRI is commonly used to visualize or rule out soft tissue structure involvement. CT is the preferred modality for identification and initial differentiation of lesions, whereas X-ray is preferred for monitoring slow-growing lesions [42]. Technetium 99m-methyl diphosphonate (99m-Tc-MDP) bone scan identifies metabolically active lesions effectively, particularly in pediatric patients above age 6. Although, it is less commonly used than CT or X-ray imaging [42,43]. 

### 1.4. Management and Treatment 

Pediatric fibrous dysplasia involving the skull base is particularly challenging to manage due to the complexity of the anatomy in the region and adjacency to neurovascular structures [9]. Surgical intervention should be considered carefully for these patients, as aggressive approaches to controlling the symptoms can entail more risk than benefit regarding complication rates, unpredictable disease course, reconstruction of significant bony defects, and overall patient status [1,9,42]. If disease status is considered active, surgical intervention should be avoided, especially in pediatric populations. This is because surgical intervention is not curative as there is no evident inhibition of continued growth, which can cause the disease to progress into adulthood [1]. Surgery should be limited to cases with immediate neurological deficits or significant deformities [1,2,42]. Indications for surgical intervention include diplopia, proptosis, compression of the optic nerve, and cranial nerve palsies. Headache is not an indication for surgery and is not necessarily resolved by surgical intervention [9]. The treatment objectives of surgery should be to prevent functional impairment, particularly regarding hearing and vision, as well as to address and reduce physical disfigurement, prevent secondary deformities, and decrease risks associated with long-term morbidity [44]. The most common reason for surgical evaluation and intervention is cosmetic. There is a great psycho-social impact on FD patients with craniofacial involvement, especially regarding decreased quality-of-life measures related to depression, anxiety, and stigma [45]. There are no guidelines on when to operate for purely cosmetic purposes [9].

Previous studies have indicated sphenoid wing involvement in nearly 40% of cases [12,22,23]. Involvement of the sphenoid wing can lead to optic nerve impingement and visual deficits. In severe cases, it can even result in complete vision loss [1,9,24,25,26,27,46,47]. Reported rates of recurrence are up to 25% after partial resection [1,9]. Although spontaneous malignant transformation is rare, radiotherapy is typically avoided because it correlates with malignant transformation [1,9,48]. Medical management with bisphosphonates has been trialed. However, it has shown little benefit and is typically limited to diffuse polyostotic patients [9,49]. The goals of medical management are pain reduction and reduced bone resorption. However, at least two studies have shown that medical management alone is ineffective for managing the symptoms [18,50]. Before surgical intervention, the goals of care should be discussed with the patient. Additionally, a multidisciplinary assessment should be completed, including evaluations by audiologists, ophthalmologists, and potentially plastic surgeons for facial asymmetry corrections. If there are hearing or vision deficits due to the progression of the disease, audiology and ophthalmology can coordinate diagnostic studies, as well as further manage and follow these symptoms [10,22]. Plastic surgeons are integral parts of the team in managing structural deformities and reconstruction, especially since esthetic deformities can take a psychological toll on patients [15,51]. Due to the complexity of managing esthetic and functional considerations, it is necessary to promote collaboration between various members of healthcare teams in an effort to provide holistic patient care and the best outcomes for patients with FD [23,51].

## 2. Methods

**Figure 1:**
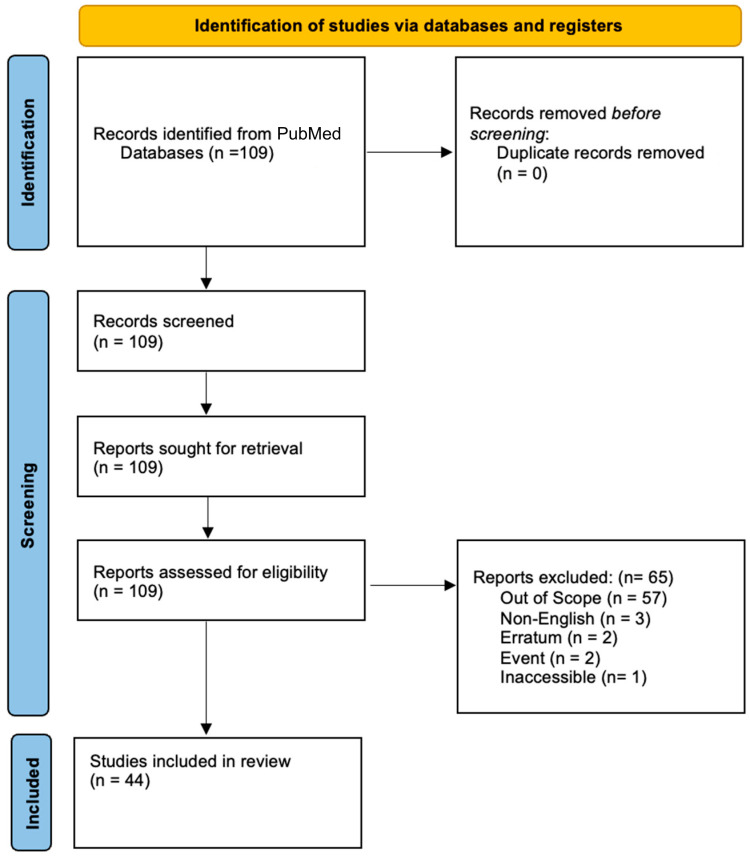
PRISMA chart summarizing review findings for pediatric fibrous dysplasia.

Articles were identified from PubMed utilizing the advanced search function and searching for the MESH terms (“pediatric” and “fibrous dysplasia” and “management”), which identifies categories and groups them based on a specific term/tag to allow for ease of filtering. The data range for included publications was from 1 January 1951 to 1 November 2024. Eligible papers must have been in the English language and accessible without additional subscriptions. Studies were excluded if they were editorials, events, errata, retracted manuscripts, instructional letters, or letters to the editor/commentary (Figure 1).

Included papers were screened for relevance and must have consisted of either a case report or a review of specifically pediatric fibrous dysplasia. Papers limited to adult presentation and management were excluded during the initial screening. The final eligible studies, which were included, are summarized in Supplementary Materials. They were reviewed for recommended guidelines regarding management, options for surgical intervention compared to medical management, and algorithms for monitoring rates of recurrence and for diagnostic radiographic imaging compared to biomarker monitoring. Of note, no paper reviewed proposed overarching guidelines for management, but rather recommended individualized approaches based on patient presentation and needs.

## 3. Results

Out of 109 articles identified for review, 44 were included for review, and 65 were excluded largely due to irrelevance (60%) (Supplementary Materials and Figure 1). Of the included articles, a total of six provided a comprehensive review of pediatric FD [1,14,15,32,41,52]. A total of four articles discussed the clear lack of guidance for the management of the pediatric population and specifically highlight the need for a multidisciplinary approach, as well as the development of monitoring algorithms for recurrence [51,53,54,55]. A total of six articles discussed medical therapeutic intervention options, which include RANK-L inhibitors and bone anti-resorptive drugs such as denosumab and zoledronate [56,57,58,59,60,61].

Most papers were case reports, with no clear guidelines established for management in the pediatric population. Rutkowski et al. in 2021 highlighted this by saying “no diagnostic and treatment algorithms, no reliable cohort epidemiological data” are found in the literature despite a clear need [54]. This stance was similar to a “lessons learned” lecture given in 2012 regarding polyostotic FD in pediatric populations, which highlighted the fact that there is no clear, effective method of management because the “degree and pattern of bone involvement [is] very individual” and this leads to limited data for evidence-based guidance [55]. Most case reports endorsed a wait-and-watch conservative approach in children due to high rates of recurrence and subsequent surgery, opting for surgical intervention only if neurological deficits, such as vision loss or hearing loss, could be mitigated or reversed with decompression and debulking [6,62,63,64,65,66]. Older papers typically endorsed a surgical management approach with radical resection preferred for those above the age of 16 due to lower recurrence rates. In patients aged less than 16, approaches were controversial, with remodeling techniques leading to high rates of relapse and subsequent surgery but less morbidity and shorter lengths of stay compared to radical resections [64,65]. Newer papers highlight the role of navigation assistance in improving outcomes or less invasive techniques such as endoscopic approaches [6,67]. There has also been an increasing role for the use of biomarkers, such as RANK-L expression leveraged for monitoring recurrence or stability of lesions, as well as diagnostic tools, such as CT imaging to help catch lesions earlier, assist with monitoring changes in density over time, and to add in surgical approaches with navigation [6,56,62,67]. Additionally, there has been increasing emphasis on the need for specialized multidisciplinary teams to aid in pain management, neuropsychiatric care, reconstruction, and optimization of surgery, as well as long-term management with imaging, biomarker monitoring, and medical therapeutics following the post-operative period [51].

## 4. Discussion

We highlight below the mentioned surgical approaches in brief. Each approach is tailored to the needs of the patients and requires lengthy discussions with multiple specialists, including plastic surgery, psychiatry or neuropsychology, neurosurgery, otolaryngology, and endocrinology, to name a few. The overall consensus of papers appears to be a trend toward conservative management for as long as possible. Surgical intervention is desired only when neurological deficit occurs and can be reversed, further deficits can be prevented, or when lesions are stable with little risk of recurrence and can be radically resected. This is typically performed when the patient is an adult and can participate more in their own care choices. There is a clear need for definitive guidelines for pediatric FD management. The current lack of clear guidelines is a byproduct of limited data due to the rarity of FD. There has been continued emphasis on creating multidisciplinary approaches and referral processes to centers that see higher case volumes of FD to assist in developing protocols and guidelines [51]. Additionally, the landscape of imaging and medical monitoring of these lesions is changing with strides in technology. Further approaches for conservative management, such as utilizing high doses of RANK-L inhibitors in individuals with high expression of RANK-L, allow for a more tailored and effective approach. Leveraging RANK-L inhibitors can delay the need for surgical intervention or even prevent regrowth after resection. The role of image-guided navigation assistance and endoscopic approaches is expanding as well, and future studies should focus on minimally invasive techniques to limit morbidity, length of stay, and maximal safe resection while reducing recurrence. Potential future studies would benefit from looking at how surgeon experiences and approaches can affect morbidity, recurrence, and patient neuropsychiatric outcomes, as this appears to be lacking in the literature. An emphasis on what types of providers should be part of a multidisciplinary team-based approach and what their role may entail would also be a beneficial part of any guideline or future study.

## 5. Limitations

Pertinent articles may have been missed in the review if they did not meet the MESH terms search criteria on PubMed: “pediatric” and “fibrous dysplasia” and “management”. Moreover, papers published in non-English languages, and which required subscriptions to access were not included. Therefore, the review may not be entirely inclusive of all the papers published on the topic. Due to the rarity of FD, there is not only limited data available, but also few articles published on the topic at hand. Further research should explore consolidating patient demographics, symptomology, management, treatment, and outcomes of pediatric fibrous dysplasia of the skull base.

## 6. Surgical Approaches

In most patients with fibrous dysplasia, the bony lesions stop growing after adolescence. Therefore, surgical intervention, especially for facial contouring and asymmetry correction, is usually better served by waiting until adulthood. Moreover, most cases of fibrous dysplasia are slow-growing and can be monitored for better surgical planning without immediate intervention. The vast majority of cases require no surgical intervention at all [1,9,68]. Lesions continue to grow in a minority of patients, more often in polyostotic disease, with an average growth period of 22.3 years versus 18.6 years in monostotic disease [68]. If surgical intervention is indicated, it is paramount to find the best approach, especially in pediatric populations. These interventions can range from conservative to radical resections and can include endoscopic and open approaches to the skull base that can involve radical osteotomies and osseous contouring [1,9,18,47,48,49,50,52,68,69]. There is currently no standardized approach for surgical management, despite attempts, and most cases are handled on a case-by-case basis [54,55,56,70].

### 6.1. Anterior Fossa Approaches

Several approaches are used to access the anterior cranial fossa. Endoscopic approaches have recently become preferred, especially in the pediatric population [9]. Limiting factors in pediatric patients include incomplete sinus pneumatization and a lack of anatomical landmarks with significant drilling. Image-based guidance is generally used to preserve normal structures and ensure complete resection [6,9]. The anterior skull base can be accessed using transfacial, transsphenoidal, frontal orbital craniotomy, zygomatic osteotomy, or anterior craniofacial approaches [1,6,42,43,46]. The risks associated with open approaches include encephalomalacia, cognitive deficits due to retraction on the developing brain, and meticulous hemostasis because pediatric patients cannot tolerate large intraoperative volume loss. Because of these concerns, endoscopic methods are often used to decompress the optic nerve [6,9,52,68,69]. Multiple approaches can be combined to address the difficulty in achieving full resection and the desire to maintain normal facial structure and support [6,9,52,68,69].

### 6.2. Middle Fossa Approaches

Approaches for accessing the middle skull base include frontotemporal with orbital or zygomatic osteotomies, anterior petroscetomy, temporal zygomatic osteomy, transsphenoidal, and preauricular infratemporal, which can require dislocation or even partial resection of the mandibular bone [1,9,52]. Common complications of anterolateral approaches include infection, meningitis, cerebrospinal fluid (CSF) leaks, cranial nerve palsies, and poor cosmetic outcomes [52]. Temporal approaches correlate with facial nerve deficits and CSF leaks, especially rhinorrhea [52,68]. Mandibular resection, or dislocation associated with infratemporal approaches, can entail complications, such as conductive hearing loss, sensory loss involving the V3 dermatome, facial nerve injury, and temporalis atrophy. Mandibular manipulation is avoided whenever possible because of the long-term detriments [1,9]. Various techniques are used in trans-sphenoidal approaches, including sublabial trans-nasal dissection, perinasal, trans-ethmoid, and endoscopic or microsurgical modifications [1,9,71,72].

### 6.3. Posterior Fossa Approaches

The posterior fossa surgical approaches include trans-petrosal, trans-labyrinthine, retrosigmoid, retro-labyrinthine, far-lateral, transcondylar, trans-cochlear, and transjugular [1,9,52,68,71]. They typically require less retraction in pediatric patients than the adult population [1]. Complications can include CSF leaks, mastoiditis due to the proximity of the mastoid air cells to the usual craniotomies in these approaches, and cranial nerve deficits [1,68,71]. The trans-cochlear approach is not recommended if hearing preservation is pertinent because it generally requires drilling the temporal bone and labyrinth [1,9]. Hearing-sparing approaches, such as infralabyrinthine and intracochlear, can be used instead, with good visualization of the petrous apex if desired [1]. The trans-jugular approach can be used to avoid mastoidectomy, as well as preserve hearing and facial nerve function, but typically requires ipsilateral occipital condyle resection to access the jugular foramen [1,9,71].

### 6.4. Postoperative Concerns

Despite surgical intervention, the most significant concern remains the continued growth of lesions, especially in incomplete resections. There appears to be less recurrence after radical resections and in patients who have reached skeletal maturity [72]. Imaging studies should be obtained for continued follow-up for potential regrowth. There is limited evidence for using alkaline phosphatase levels to monitor recurrence, though one study has shown it to increase drastically in patients just before recurrence [9,72].

## 7. Conclusions

Fibrous dysplasia is commonly a benign, insidious bony lesion with limited functional implications. Still, when there is craniofacial involvement, growth can cause drastic neurological deficits, such as optic nerve compression, that warrant urgent surgical intervention. Craniofacial involvement occurs in pediatric populations and requires a multidisciplinary approach to optimize patient outcomes. The most common reason for surgical intervention is cosmetic, and many surgical techniques, such as bone contouring, are used. However, when there is sphenoid or sellar involvement, endoscopic approaches or even radical resections can be the best option for preventing the recurrence of lesions. Combined approaches can often optimize patient safety and outcomes. Careful follow-up with imaging is necessary as there can be a high rate of recurrence or, rarely, malignant transformation. The current literature lacks clear guidance on management, especially with regard to surgical intervention or recurrence monitoring algorithms.

## Figures and Tables

**Table 1:** Summary of bone involvement in monostotic, polyostotic, and McCune-Albright syndrome fibrous dysplasia subtypes.

Study	FD Type	Population	N	Maxilla	Orbit	Mandible	Frontal	Temporal	Sphenoid	Occipital	Ethmoid	Zygomatic	Parietal	Clivus	Nasal	Skull Base
*Prevalence of Different Forms and Involved Bones of Craniofacial Fibrous Dysplasia (Yang et al., 2017) [12]*	M	Adult and pediatric	289	28%	27%	25%	22%	12%	2%	3%	5%	9%	5%			
	P	Adult and pediatric	163	30%		21%	25%	11%	26%	7%	23%	27%	8%	4%	3%	
	MAS	Adult and pediatric	8	4%		4%			5%							
*Polyostotic Fibrous Dysplasia with and Without McCune-Albright Syndrome-Clinical Features in a Nordic Pediatric Cohort (Utriainen et al., 2018) [23]*	MAS/P	Pediatric	16 (8 MAS)	56%	50%	25%	38%	25%	44%	19%	13%	6%	31%			19%
*Management Strategies of Fibrous Dysplasia Involving the Paranasal Sinus and the Adjacent Skull Base (Shi et al., 2022) [6]*	M	Adult and pediatric	19	5%			5%		58%		32%					
	P	Adult and pediatric	3					33%	100%		67%					
	MAS	Adult and pediatric	1	100%			100%	100%	100%		100%	100%				
M = Monostotic P = Polyostotic MAS = McCune-Albright Syndrome																

## Data Availability

Data is contained within the article or Supplementary Material.

## Supplementary Materials

The following supporting information can be downloaded at: https://www.mdpi.com/article/10.3390/brainsci14121210/s1.

## References

[1] Tsai E.C., Santoreneos S., Rutka J.T. (2002). Tumors of the skull base in children: Review of tumor types and management strategies. Neurosurg. Focus.

[2] Lee J.S., FitzGibbon E.J., Chen Y.R., Kim H., Lustig L., Akintoye S., Collins M., Kaban L. (2012). Clinical guidelines for the management of craniofacial fibrous dysplasia. Orphanet J. Rare Dis..

[3] Riminucci M., Fisher L.W., Shenker A., Spiegel A.M., Bianco P., Gehron Robey P. (1997). Fibrous dysplasia of bone in the McCune-Albright syndrome: Abnormalities in bone formation. Am. J. Pathol..

[4] Schwindinger W.F., Francomano C.A., Levine M.A. (1992). Identification of a mutation in the gene encoding the alpha subunit of the stimulatory G protein of adenylyl cyclase in McCune-Albright syndrome. Proc. Natl. Acad. Sci. USA.

[5] Weinstein L.S., Shenker A., Gejman P.V., Merino M.J., Friedman E., Spiegel A.M. (1991). Activating mutations of the stimulatory G protein in the McCune–Albright syndrome. N. Engl. J. Med..

[6] Shi L.-L., Xiong P., Zhen H.-T. (2022). Management Strategies of Fibrous Dysplasia Involving the Paranasal Sinus and the Adjacent Skull Base. Ear Nose Throat J..

[7] Zhao X., Deng P., Iglesias-Bartolome R., Amornphimoltham P., Steffen D.J., Jin Y., Molinolo A.A., de Castro L.F., Ovejero D., Yuan Q. (2018). Expression of an active Gα_s_ mutant in skeletal stem cells is sufficient and necessary for fibrous dysplasia initiation and maintenance. Proc. Natl. Acad. Sci. USA.

[8] Raimondo D., Remoli C., Astrologo L., Burla R., La Torre M., Vernì F., Tagliafico E., Corsi A., Del Giudice S., Persichetti A. (2020). Changes in gene expression in human skeletal stem cells transduced with constitutively active Gsα correlates with hallmark histopathological changes seen in fibrous dysplastic bone. PLoS ONE.

[9] Wilson M., Snyderman C. (2018). Fibro-Osseous Lesions of the Skull Base in the Pediatric Population. J. Neurol. Surg. Part B Skull Base.

[10] Parekh S.G., Donthineni-Rao R., Ricchetti E., Lackman R.D. (2004). Fibrous dysplasia. J. Am. Acad. Orthop. Surg..

[11] Dumitrescu C.E., Collins M.T. (2008). McCune-Albright syndrome. Orphanet J. Rare Dis..

[12] Yang L., Wu H., Lu J., Teng L. (2017). Prevalence of Different Forms and Involved Bones of Craniofacial Fibrous Dysplasia. J. Craniofac. Surg..

[13] Park J.W., Jung J.H., Park S.J., Lim S.Y. (2020). Evaluation of natural growth rate and recommended age for shaving procedure by volumetric analysis of craniofacial fibrous dysplasia. Head Neck.

[14] Boyce A.M., Burke A., Peck C.C., DuFresne C.R., Lee J.S., Collins M.T. (2016). Surgical Management of Polyostotic Craniofacial Fibrous Dysplasia: Long-Term Outcomes and Predictors for Postoperative Regrowth. Plast. Reconstr. Surg..

[15] Gun Z.H., Arif A., Boyce A.M. (2024). Fibrous dysplasia in children and its management. Curr. Opin. Endocrinol. Diabetes Obes..

[16] Amit M., Fliss D.M., Gil Z. (2011). Fibrous dysplasia of the sphenoid and skull base. Otolaryngol. Clin. N. Am..

[17] Schreiber A., Villaret A.B., Maroldi R., Nicolai P. (2012). Fibrous dysplasia of the sinonasal tract and adjacent skull base. Curr. Opin. Otolaryngol. Head Neck Surg..

[18] Edgerton M.T., Persing J.A., Jane J.A. (1985). The surgical treatment of fibrous dysplasia. With emphasis on recent contributions from cranio-maxillo-facial surgery. Ann. Surg..

[19] Henry A. (1969). Monostotic fibrous dysplasia. J. Bone Jt. Surg. Br. Vol..

[20] Sherman N.H., Rao V.M., Brennan R.E., Edeiken J. (1982). Fibrous dysplasia of the facial bones and mandible. Skelet. Radiol..

[21] Valentini V., Cassoni A., Marianetti T.M., Terenzi V., Fadda M.T., Iannetti G. (2009). Craniomaxillofacial fibrous dysplasia: Conservative treatment or radical surgery? A retrospective study on 68 patients. Plast. Reconstr. Surg..

[22] Lee J.S., FitzGibbon E., Butman J.A., Dufresne C.R., Kushner H., Wientroub S., Robey P.G., Collins M.T. (2002). Normal vision despite narrowing of the optic canal in fibrous dysplasia. N. Engl. J. Med..

[23] Utriainen P., Valta H., Björnsdottir S., Mäkitie O., Horemuzova E. (2018). Polyostotic Fibrous Dysplasia With and Without McCune-Albright Syndrome-Clinical Features in a Nordic Pediatric Cohort. Front. Endocrinol..

[24] Lustig L.R., Holliday M.J., McCarthy E.F., Nager G.T. (2001). Fibrous dysplasia involving the skull base and temporal bone. Arch. Otolaryngol. Head Neck Surg..

[25] Hart E.S., Kelly M.H., Brillante B., Chen C.C., Ziran N., Lee J.S., Feuillan P., I Leet A., Kushner H., Robey P.G. (2007). Onset, progression, and plateau of skeletal lesions in fibrous dysplasia and the relationship to functional outcome. J. Bone Miner. Res..

[26] Salmasi V., Blitz A.M., Ishii M., Gallia G.L. (2011). Expanded endonasal endoscopic approach for resection of a large skull base aneurysmal bone cyst in a pediatric patient with extensive cranial fibrous dysplasia. Child’s Nerv. Syst..

[27] Stapleton A.L., Tyler-Kabara E.C., Gardner P.A., Snyderman C.H. (2015). Endoscopic endonasal surgery for benign fibro-osseous lesions of the pediatric skull base. Laryngoscope.

[28] Sweeney K., Kaban L.B. (2020). Natural History and Progression of Craniofacial Fibrous Dysplasia: A Retrospective Evaluation of 114 Patients From Massachusetts General Hospital. J. Oral Maxillofac. Surg..

[29] Akintoye S.O., Lee J.S., Feimster T., Booher S., Brahim J., Kingman A., Riminucci M., Robey P.G., Collins M.T. (2003). Dental characteristics of fibrous dysplasia and McCune-Albright syndrome. Oral Surg. Oral Med. Oral Pathol. Oral Radiol. Endod..

[30] Kapitanov D., Kostousova A., Nersesyan M., Vicheva D., Lenchyk L.V., Georgiev K. (2019). Sinonasal fibrous dysplasia: Our 10-years experience. J. IMAB–Annu. Proceeding Sci. Pap..

[31] DeKlotz T.R., Kim H.J., Kelly M., Collins M.T. (2013). Sinonasal disease in polyostotic fibrous dysplasia and McCune–Albright Syndrome. Laryngoscope.

[32] Szymczuk V., Taylor J., Boyce A.M. (2023). Craniofacial Fibrous Dysplasia: Clinical and Therapeutic Implications. Curr. Osteoporos. Rep..

[33] Bibby K., McFadzean R. (1994). Fibrous dysplasia of the orbit. Br. J. Ophthalmol..

[34] Ricalde P., Horswell B.B. (2001). Craniofacial fibrous dysplasia of the fronto-orbital region: A case series and literature review. J. Oral Maxillofac. Surg..

[35] Boyce A.M., Brewer C., DeKlotz T.R., Zalewski C.K., King K.A., Collins M.T., Kim H.J. (2018). Association of Hearing Loss and Otologic Outcomes With Fibrous Dysplasia. JAMA Otolaryngol. Head Neck Surg..

[36] Frisch C.D., Carlson M.L., Kahue C.N., Pelosi S., Haynes D.S., Lane J.I., Neff B.A., Link M.J., Driscoll C.L.W. (2015). Fibrous dysplasia of the temporal bone: A review of 66 cases. Laryngoscope.

[37] Burke A.B., Collins M.T., Boyce A.M. (2017). Fibrous dysplasia of bone: Craniofacial and dental implications. Oral Dis..

[38] Cutler C.M., Lee J.S., Butman J.A., FitzGibbon E.J., Kelly M.H., Brillante B.A., Feuillan P., Robey P.G., DuFresne C.R., Collins M.T. (2006). Long-term outcome of optic nerve encasement and optic nerve decompression in patients with fibrous dysplasia: Risk factors for blindness and safety of observation. Neurosurgery.

[39] Pan K.S., Heiss J.D., Brown S.M., Collins M.T., Boyce A.M. (2018). Chiari I Malformation and Basilar Invagination in Fibrous Dysplasia: Prevalence, Mechanisms, and Clinical Implications. J. Bone Miner. Res..

[40] El-Mofty S.K. (2014). Fibro-osseous lesions of the craniofacial skeleton: An update. Head Neck Pathol..

[41] Hartley I., Zhadina M., Collins M.T., Boyce A.M. (2019). Fibrous Dysplasia of Bone and McCune–Albright Syndrome: A Bench to Bedside Review. Calcif. Tissue Int..

[42] Kushchayeva Y.S., Kushchayev S.V., Glushko T.Y., Tella S.H., Teytelboym O.M., Collins M.T., Boyce A.M. (2018). Fibrous dysplasia for radiologists: Beyond ground glass bone matrix. Insights Into Imaging.

[43] Collins M.T., Kushner H., Reynolds J.C., Chebli C., Kelly M.H., Gupta A., Brillante B., I Leet A., Riminucci M., Robey P.G. (2005). An instrument to measure skeletal burden and predict functional outcome in fibrous dysplasia of bone. J. Bone Miner. Res..

[44] Javaid M.K., Boyce A., Appelman-Dijkstra N., Ong J., Defabianis P., Offiah A., Arundel P., Shaw N., Pos V.D., Underhil A. (2019). Best practice management guidelines for fibrous dysplasia/McCune-Albright syndrome: A consensus statement from the FD/MAS international consortium. Orphanet J. Rare Dis..

[45] Konradi A. (2022). Fibrous dysplasia patients with and without craniofacial involvement report reduced quality of life inclusive of stigma, depression, and anxiety. Chronic Illn..

[46] Snyderman C.H., Lavigne P. (2020). Benign Tumors of the Anterior Cranial Base. Adv Otorhinolaryngol..

[47] Manjila S., Zender C.A., Weaver J., Rodgers M., Cohen A.R. (2013). Aneurysmal bone cyst within fibrous dysplasia of the anterior skull base: Continued intracranial extension after endoscopic resections requiring craniofacial approach with free tissue transfer reconstruction. Child’s Nerv. Syst..

[48] Di Rocco C., Marchese E., Velardi F. (1992). Fibrous dysplasia of the skull in children. Pediatr. Neurosurg..

[49] Chapurlat R.D. (2006). Medical therapy in adults with fibrous dysplasia of bone. J. Bone Miner. Res..

[50] Béquignon E., Cardinne C., Lachiver X., Wagner I., Chabolle F., Baujat B. (2013). Craniofacial fibrous dysplasia surgery: A functional approach. Eur. Ann. Otorhinolaryngol. Head Neck Dis..

[51] Meier M.E., Hagelstein-Rotman M., van de Ven A.C., Van der Geest I.C.M., Donker O., Pichardo S.E.C., Muller P.C.E.H., van der Meeren S.W., Dorleijn D.M.J., Winter E.M. (2022). A multidisciplinary care pathway improves quality of life and reduces pain in patients with fibrous dysplasia/McCune-Albright syndrome: A multicenter prospective observational study. Orphanet J. Rare Dis..

[52] Denadai R., Raposo-Amaral C.A., Marques F.F., Ghizoni E., Buzzo C.L., Raposo-Amaral C.E. (2016). Strategies for the Optimal Individualized Surgical Management of Craniofacial Fibrous Dysplasia. Ann. Plast. Surg..

[53] Golden E., van der Heijden H., Ren B., Randall E.T., A Drubach L., Shah N., Cay M., Ebb D., Kaban L.B., Peacock Z.S. (2024). Phenotyping Pain in Patients With Fibrous Dysplasia/McCune-Albright Syndrome. J. Clin. Endocrinol. Metab..

[54] Rutkowski M., Niewinska K. (2021). The Epidemiology of Benign Proliferative Processes of the Skeletal System in Children. Int. J. Environ. Res. Public Health.

[55] Stanton R.P., Ippolito E., Springfield D., Lindaman L., Wientroub S., Leet A. (2012). The surgical management of fibrous dysplasia of bone. Orphanet J. Rare Dis..

[56] Raborn L.N., Burke A.B., Ebb D.H., Collins M.T., Kaban L.B., Boyce A.M. (2021). Denosumab for craniofacial fibrous dysplasia: Duration of efficacy and post-treatment effects. Osteoporos. Int..

[57] A Vanderniet J., Szymczuk V., Högler W., Beck-Nielsen S.S., Uday S., Merchant N., Crane J.L., Ward L.M., Boyce A.M., Munns C.F. (2024). Management of RANKL-mediated Disorders With Denosumab in Children and Adolescents: A Global Expert Guidance Document. J. Clin. Endocrinol. Metab..

[58] Leet A.I., Collins M.T. (2007). Current approach to fibrous dysplasia of bone and McCune–Albright syndrome. J. Child. Orthop..

[59] Zacharin M. (2005). Paediatric management of endocrine complications in McCune-Albright syndrome. J. Pediatr. Endocrinol. Metab..

[60] Bharwani N., Rathod P., Salunke A.A., Patel D., Tripathi U., Varun M., Krishana G., Dave D., Patel K., Sharma M. (2024). Fibrous Dysplasia Involving Cranio-Facial Region Treated with Zolendronic Acid: A Single Institutional Experience and Review of Literature. Indian J. Otolaryngol. Head Neck Surg..

[61] Jayant S.S., Walia R., Gupta R., Pal R., Chaudhary S., Agrawal K., Rastogi A., Bhattacharya A., Dutta P., Bhadada S.K. (2023). Autonomous growth hormone secretion due to McCune Albright syndrome in paediatric age group: An ominous triad. Endocrine.

[62] Valentini V., Cassoni A., Terenzi V., Della Monaca M., Fadda M., Zadeh O.R., Raponi I., Anelli A., Iannetti G. (2017). Our experience in the surgical management of craniofacial fibrous dysplasia: What has changed in the last 10 years?. Acta Otorhinolaryngol. Ital..

[63] Mierzwiński J., Kosowska J., Tyra J., Haber K., Drela M., Paczkowski D., Burduk P. (2018). Different clinical presentation and management of temporal bone fibrous dysplasia in children. World J. Surg. Oncol..

[64] Giordano F., Serio P., Savasta S., Oliveri G., Genitori L. (2006). Craniofacial surgery in fibrous dysplasia. J. Pediatr. Endocrinol. Metab..

[65] Elwy R., Gokden M., Cai R. (2018). Emergency Optic Canal Decompression for Vision Salvage in Fibrous Dysplasia. World Neurosurg..

[66] Mehta D., Clifton N., McClelland L., Jones N.S. (2006). Paediatric fibro-osseous lesions of the nose and paranasal sinuses. Int. J. Pediatr. Otorhinolaryngol..

[67] Best D.L., Lee K.C., Reynolds R.M., Piccillo E., Behar P., Markiewicz M.R. (2024). Contemporary Surgical Management of Craniofacial Fibrous Dysplasia Using Computer-Assisted Surgery and Intraoperative Navigation. J. Craniofac. Surg..

[68] Kusano T., Hirabayashi S., Eguchi T., Sugawara Y. (2009). Treatment strategies for fibrous dysplasia. J. Craniofac. Surg..

[69] Jeyaraj P. (2019). Histological Diversity, Diagnostic Challenges, and Surgical Treatment Strategies of Fibrous Dysplasia of Upper and Mid-Thirds of the Craniomaxillofacial Complex. Ann. Maxillofac. Surg..

[70] Boyce A.M., Kelly M.H., Brillante B.A., Kushner H., Wientroub S., Riminucci M., Bianco P., Robey P.G., Collins M.T. (2014). A randomized, double blind, placebo-controlled trial of alendronate treatment for fibrous dysplasia of bone. J. Clin. Endocrinol. Metab..

[71] Wei Y.-T., Jiang S., Cen Y. (2010). Fibrous dysplasia of skull. J. Craniofac. Surg..

[72] Park B.Y., Cheon Y.W., Kim Y.O., Pae N.S., Lee W.J. (2010). Prognosis for craniofacial fibrous dysplasia after incomplete resection: Age and serum alkaline phosphatase. Int. J. Oral Maxillofac. Surg..

