# Supplementary material for: Pediatric Fibrous Dysplasia of the Skull Base: Update on Management and Treatment
Source: Brain Sci. 2024 Nov 29;14(12):1210. doi: 10.3390/brainsci14121210 (PMC11674249; doi:10.3390/brainsci14121210)
Supplement: Supplementary file 1 [file brainsci-14-01210-s001.zip › brainsci-3285507-supplementary.pdf]

brain sciences

MDPI

Supplementary Table 1

Title	Journal/Book	Authors	Publication Year
A multidisciplinary care pathway improves quality of life and reduces pain in patients with fibrous dysplasia/McCune-Albright syndrome: a multicenter prospective observational study	Orphanet Journal of Rare Diseases	Meier ME, Hagelstein-Rotman M, van de Ven AC, Van der Geest ICM, Donker O, Pichardo SEC, Hissink Muller PCE, van der Meeren SW, Dorleijn DMJ, Winter EM, van de Sande MAJ, Appelman-Dijkstra NM.	2022
A Rare Case of Fibrous Dysplasia Presenting With Facial Swelling and Craniofacial Deformity in a 13-Year-Old Girl	Cureus Journal of Medical Science	Dhabalia R, Kashikar SV, Parihar P, Naseri S.	2024
Autonomous growth hormone secretion due to McCune Albright syndrome in paediatric age group: an ominous triad	Endocrine	Jayant SS, Walia R, Gupta R, Pal R, Chaudhary S, Agrawal K, Rastogi A, Bhattacharya A, Dutta P, Bhadada SK, Bhansali A.	2023
Benign skull and subdural lesions in patients with prior medulloblastoma therapy	Child's Nervous System	Kaneva K, Wadhwani N, DiPatri AJ Jr, Palasis S, Goldman S, Aw-Zoretic J.	2021
Changing referral patterns to a designated craniofacial centre over a four-year period	Journal of Plastic, Reconstructive & Aesthetic Surgery	White N, Warner RM, Noons P, McAlister EM, Solanki G, Nishikawa H, Dover MS.	2010
Clinical Characteristics and Management of Patients With McCune-Albright Syndrome With GH Excess and Precocious Puberty: A Case Series and Literature Review	Frontiers in Endocrinology	Zhai X, Duan L, Yao Y, Xing B, Deng K, Wang L, Feng F, Liang Z, You H, Yang H, Lu L, Chen S, Wang R, Pan H, Zhu H.	2021
Comprehensive management of malocclusion in maxillary fibrous dysplasia: A case report	World Journal of Clinical Cases	Kaur H, Mohanty S, Kochhar GK, Iqbal S, Verma A, Bhasin R, Kochhar AS.	2021
Contemporary Surgical Management of Craniofacial Fibrous Dysplasia Using Computer-Assisted Surgery and Intraoperative Navigation	Journal of Craniofacial Surgery	Best DL, Lee KC, Reynolds RM, Piccillo E, Behar P, Markiewicz MR.	2024

Craniofacial Fibrous Dysplasia: Clinical and Therapeutic Implications	Current Osteoporosis Reports	Szymczuk V, Taylor J, Boyce AM.	2023
Craniofacial fibrous dysplasia. conservative surgical management. Review of literature and report of a case	Folia Medica	Anastassov YC, Anastassov GE, Lumerman HS, Simov R, Shipkov CD, Mihailov TA, Traikova N.	2004
Craniofacial surgery in fibrous dysplasia	Journal of Pediatric Endocrinology and Metabolism	Giordano F, Serio P, Savasta S, Oliveri G, Genitori L.	2006
Current approach to fibrous dysplasia of bone and McCune-Albright syndrome	Journal of Children's Orthopaedics	Leet AI, Collins MT.	2007
Denosumab for craniofacial fibrous dysplasia: duration of efficacy and post-treatment effects	Osteoporosis International	Raborn LN, Burke AB, Ebb DH, Collins MT, Kaban LB, Boyce AM.	2021
Diagnosis and Treatment of Pediatric Primary Jaw Lesions at Massachusetts General Hospital	Journal of Oral and Maxillofacial Surgery	Zhang J, Troulis MJ, August M.	2021
Different clinical presentation and management of temporal bone fibrous dysplasia in children	World Journal of Surgical Oncology	Mierzwiński J, Kosowska J, Tyra J, Haber K, Drela M, Paczkowski D, Burduk P.	2018
Emergency Optic Canal Decompression for Vision Salvage in Fibrous Dysplasia	World Neurosurgery	Elwy R, Gokden M, Cai R.	2018
Endoscopic surgery for fibrous dysplasia of the sinonasal tract in pediatric patients	International Journal of Pediatric Otorhinolaryngology	Berlucchi M, Salsi D, Farina D, Nicolai P.	2005
Expression of Beta-Catenin, Cadherins and P-Runx2 in Fibro-Osseous Lesions of the Jaw: Tissue Microarray Study	Biomolecules	Pannone G, Nocini R, Santoro A, Spirito F, Nocini PF, Papagerakis S, Franceschi RT, Di Domenico M, Di Carlo A, Danelia N, Lo Muzio L.	2022
Fibrous Dysplasia	Endotext	Boyce AM.	2000
Fibrous dysplasia in children and its management	Current Opinions on Endocrinology, Diabetes, and Obsesity	Gun ZH, Arif A, Boyce AM.	2024
Fibrous Dysplasia Involving Cranio-Facial Region Treated with Zolendronic Acid: A	Indian Journal of Otolaryngology and Head & Neck Surgery	Bharwani N, Rathod P, Salunke AA, Patel D, Tripathi U, Varun M, Krishana G,	2024

Single Institutional Experience and Review of Literature		Dave D, Patel K, Sharma M, Puj K, Aron J, Bhalerao R, Shah K, Deshmukh S, Pandya S.	
Fibrous Dysplasia of Bone and McCune-Albright Syndrome: A Bench to Bedside Review	Calcified Tissue International	Hartley I, Zhadina M, Collins MT, Boyce AM.	2019
Fibrous dysplasia: rare manifestation in the temporal bone	Brazilian Journal of Otorhinolaryngology	Pontes-Madruga TC, Filgueiras HVC, Silva DMSD, Silva LSD, Testa JRG.	2022
Frontal bone fibrous dysplasia in a 6-months-old boy: A distinctive entity	La Pediatria Medica e Chirurgica	Anastasiadis K, Lambropoulos V, Tsoleka K, Kepertis C, Mouravas V, Spyridakis I.	2021
Incidence of biopsy-proven bone tumors in children: a report based on the Dutch pathology registration "PALGA"	Journal of Pediatric Orthopaedics	van den Berg H, Kroon HM, Slaar A, Hogendoorn P.	2008
Low-grade bone lesions in survivors of childhood medulloblastoma/primitive neuroectodermal tumor	Academic Radiology	Koral K, Roy D, Timmons CF, Gargan L, Bowers DC.	2012
Management of RANKL-mediated Disorders With Denosumab in Children and Adolescents: A Global Expert Guidance Document	The Journal of Clinical Endocrinology and Metabolism	Vanderniet JA, Szymczuk V, Högler W, Beck-Nielsen SS, Uday S, Merchant N, Crane JL, Ward LM, Boyce AM, Munns CF.	2024
Management Strategies of Fibrous Dysplasia Involving the Paranasal Sinus and the Adjacent Skull Base	Ear Nose Throat Journal	Shi LL, Xiong P, Zhen HT.	2022
Mandibular mass in pediatric patient: a diagnostic dilemma	Journal of Indian Society of Pedodontics and Preventive Dentistry	Ranadive PA, Deshpande MD, Ingole SN, Ankush C.	2012
McCune-Albright syndrome, natural history and multidisciplinary management in a series of 14 pediatric cases	The Annales d'Endocrinologie	Agopiantz M, Journeau P, Lebon-Labich B, Sorlin A, Cuny T, Weryha G, Leheup B.	2016
McCune-Albright syndrome: a case report and review of the literature	International Journal of Pediatric Otorhinolaryngology	Bolger WE, Ross AT.	2002

Medication Management of Selected Pathological Jaw Lesions	Oral and Maxillofacial Surgery Clinics of North America	Fan Y, Glied A.	2022
Our experience in the surgical management of craniofacial fibrous dysplasia: what has changed in the last 10 years?	ACTA Otorhinolaryngologica Italica	Valentini V, Cassoni A, Terenzi V, Della Monaca M, Fadda MT, Rajabtork Zadeh O, Raponi I, Anelli A, Iannetti G.	2017
Paediatric fibro-osseous lesions of the nose and paranasal sinuses	International Journal of Pediatric Otorhinolaryngology	Mehta D, Clifton N, McClelland L, Jones NS.	2006
Paediatric management of endocrine complications in McCune-Albright syndrome	Journal of Pediatric Endocrinology and Metabolism	Zacharin M.	2005
Paediatric paranasal sinus fibrous dysplasia	Tropical Doctor	Adeyemo AA, Ogunkeyede SA, Daniel A, Lasisi AO.	2023
Pediatric Benign Fibro-Osseous Lesions of the Nose and Paranasal Sinuses: A Tertiary Hospital Experience	International Journal of Otolaryngology	Al Arfaj D, Alenzi HL, Almomen A, Bakri M.	2022
Periorbital inflammation associated with craniofacial fibrous dysplasia: Report of three cases and review of the literature	Bone	Theng EH, German A, Pan KS, Isaac S, Boyce AM, Collins MT.	2021
Phenotyping Pain in Patients With Fibrous Dysplasia/McCune-Albright Syndrome	The Journal of Clinical Endocrinology and Metabolism	Golden E, van der Heijden H, Ren B, Randall ET, Drubach LA, Shah N, Cay M, Ebb D, Kaban LB, Peacock ZS, Boyce AM, Mannstadt M, Upadhyay J.	2024
Sphenoid masses in children: radiologic differential diagnosis with pathologic correlation	American Journal of Neuroradiology	Lui YW, Dasari SB, Young RJ.	2011
Strategies for the Optimal Individualized Surgical Management of Craniofacial Fibrous Dysplasia	Annals of Plastic Surgery	Denadai R, Raposo-Amaral CA, Marques FF, Ghizoni E, Buzzo CL, Raposo-Amaral CE.	2016
The Epidemiology of Benign Proliferative Processes of the Skeletal System in Children	International Journal of Environmental Research and Public Health	Rutkowski M, Niewinska K.	2021

The surgical management of fibrous dysplasia of bone	Orphanet Journal of Rare Diseases	Stanton RP, Ippolito E, Springfield D, Lindaman L, Wientroub S, Leet A.	2012
Tumors of the skull base in children: review of tumor types and management strategies	Neurosurgical Focus	Tsai EC, Santoreneos S, Rutka JT.	2002

Disclaimer/Publisher's Note: The statements, opinions and data contained in all publications are solely those of the individual author(s) and contributor(s) and not of MDPI and/or the editor(s). MDPI and/or the editor(s) disclaim responsibility for any injury to people or property resulting from any ideas, methods, instructions or products referred to in the content.
